# Supplementary material for: Putting Laccase Gene Differences on Genomic Level into Context: An Analysis of Botrytis cinerea Strains from Grapes
Source: Microorganisms. 2025 Feb 21;13(3):483. doi: 10.3390/microorganisms13030483 (PMC11945579; doi:10.3390/microorganisms13030483)
Supplement: Supplementary file 1 [file microorganisms-13-00483-s001.zip › microorganisms-3466528-supplementary.pdf]

microorganisms

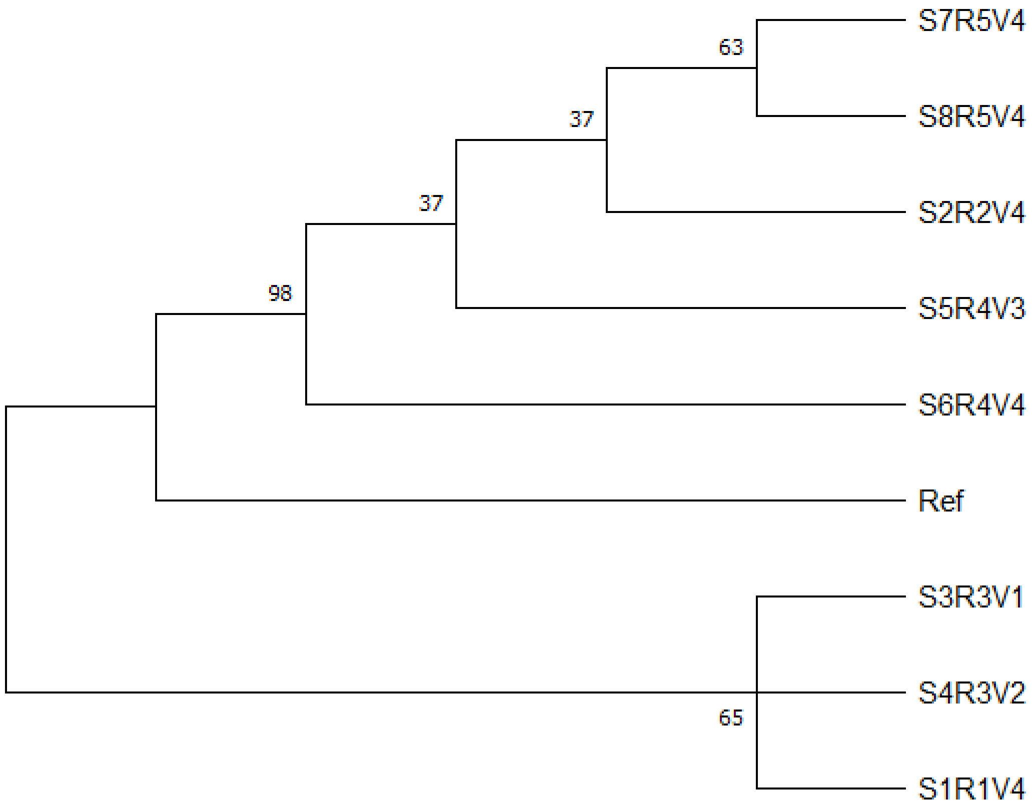

Figure S1. Maximum likelihood tree of *Lac 1* Gene sequences of *Botrytis cinerea* combined and compared for every strain. The strains selected were from different regions and vintages. Bootstrap values were calculated from 1000 iterations and are displayed in %.

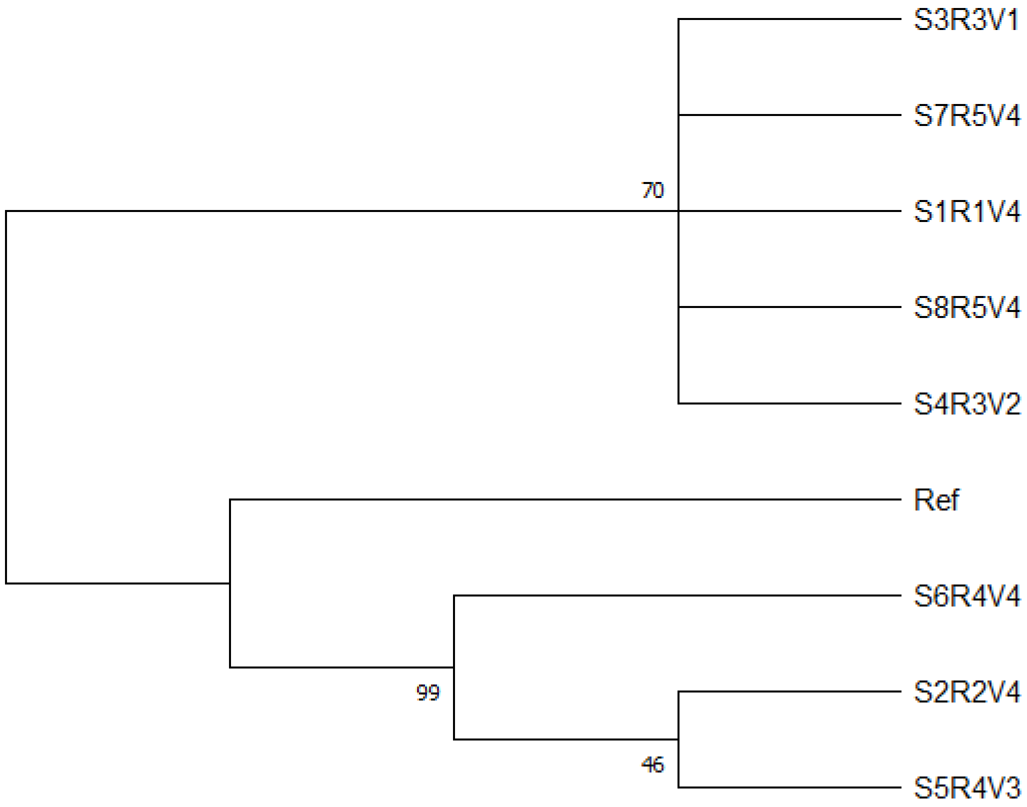

Figure S2. Maximum likelihood tree of *Lac 2* Gene sequences of *Botrytis cinerea* combined and compared for every strain. The strains selected were from different regions and vintages. Bootstrap values were calculated from 1000 iterations and are displayed in %.

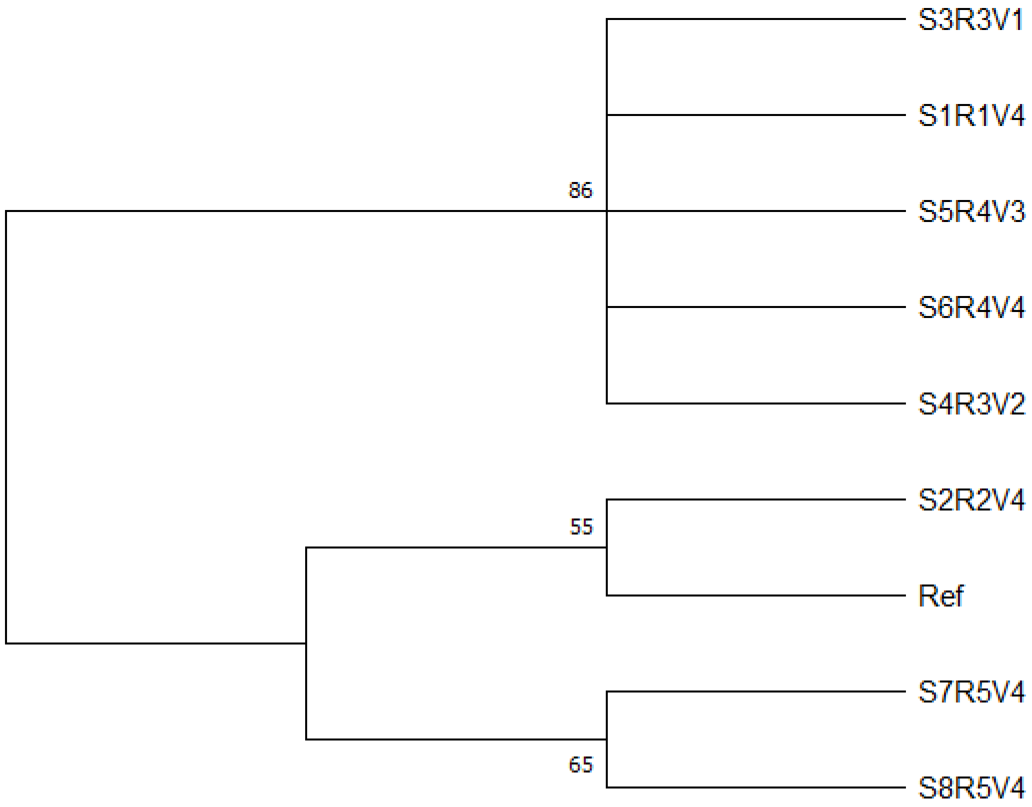

Figure S3. Maximum likelihood tree of *Lac 3* Gene sequences of *Botrytis cinerea* combined and compared for every strain. The strains selected were from different regions and vintages. Bootstrap values were calculated from 1000 iterations and are displayed in %.

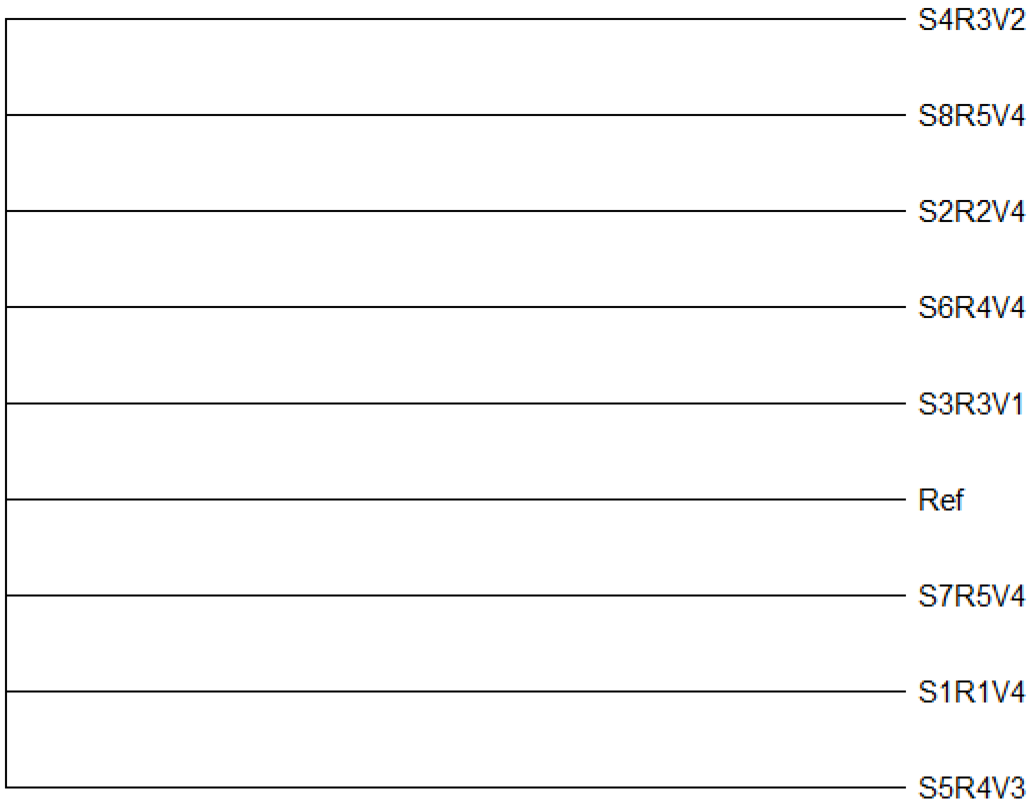

Figure S4. Maximum likelihood tree of *Lac 4* Gene sequences of *Botrytis cinerea* combined and compared for every strain. The strains selected were from different regions and vintages. Bootstrap values were calculated from 1000 iterations and are displayed in %.

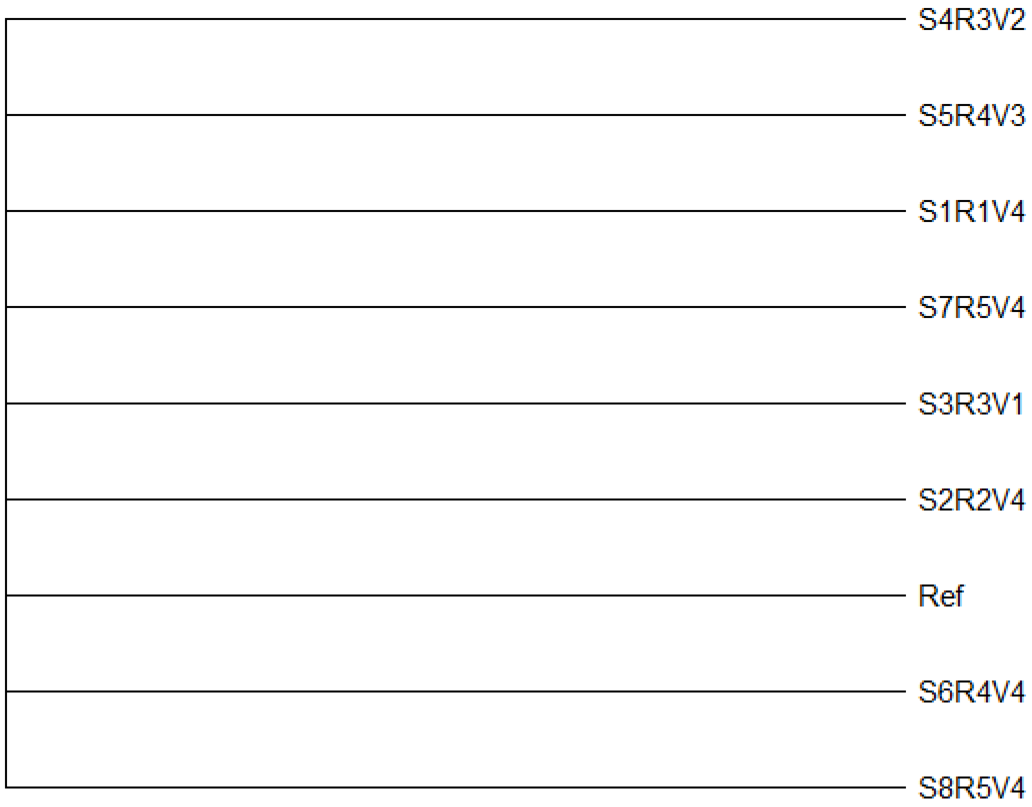

Figure S5. Maximum likelihood tree of *Lac 5* Gene sequences of *Botrytis cinerea* combined and compared for every strain. The strains selected were from different regions and vintages. Bootstrap values were calculated from 1000 iterations and are displayed in %.

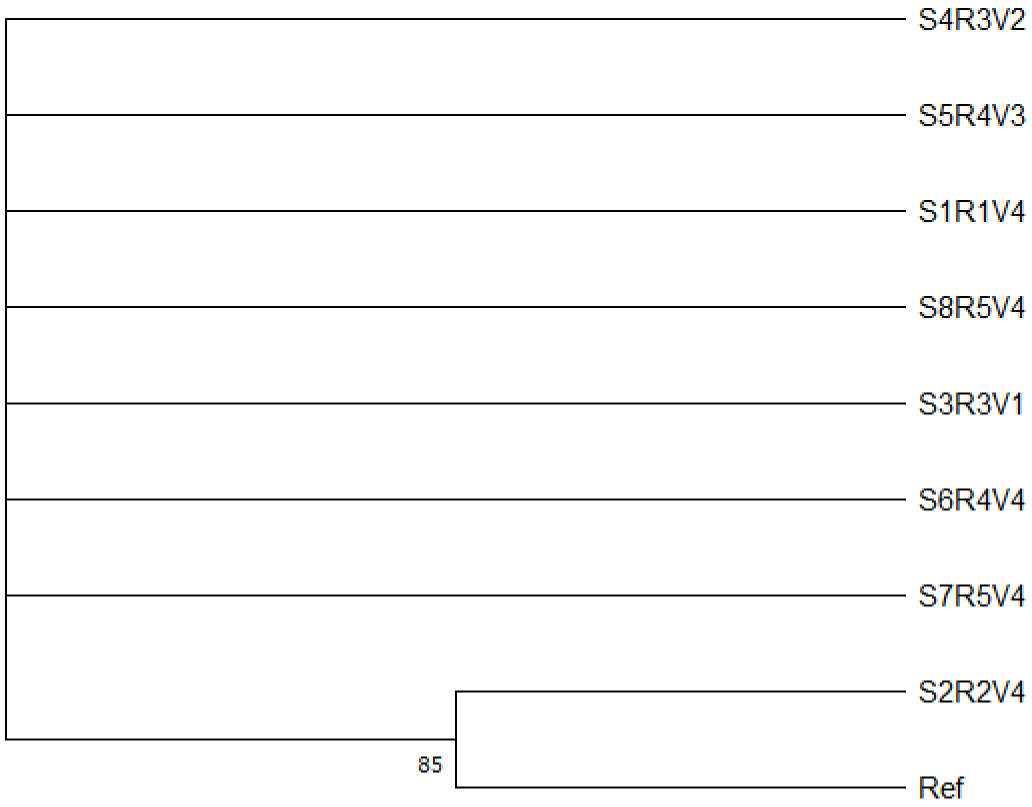

Figure S6. Maximum likelihood tree of *Lac 6* Gene sequences of *Botrytis cinerea* combined and compared for every strain. The strains selected were from different regions and vintages. Bootstrap values were calculated from 1000 iterations and are displayed in %.

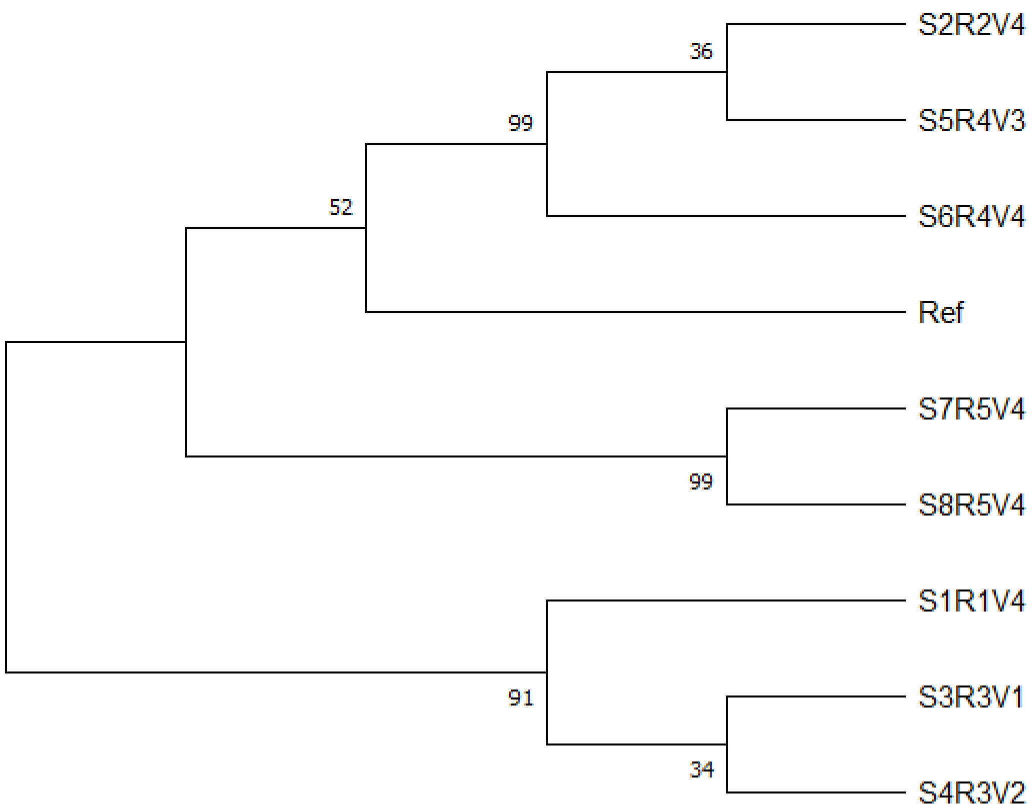

Figure S7. Maximum likelihood tree of *Lac 7* Gene sequences of *Botrytis cinerea* combined and compared for every strain. The strains selected were from different regions and vintages. Bootstrap values were calculated from 1000 iterations and are displayed in %.

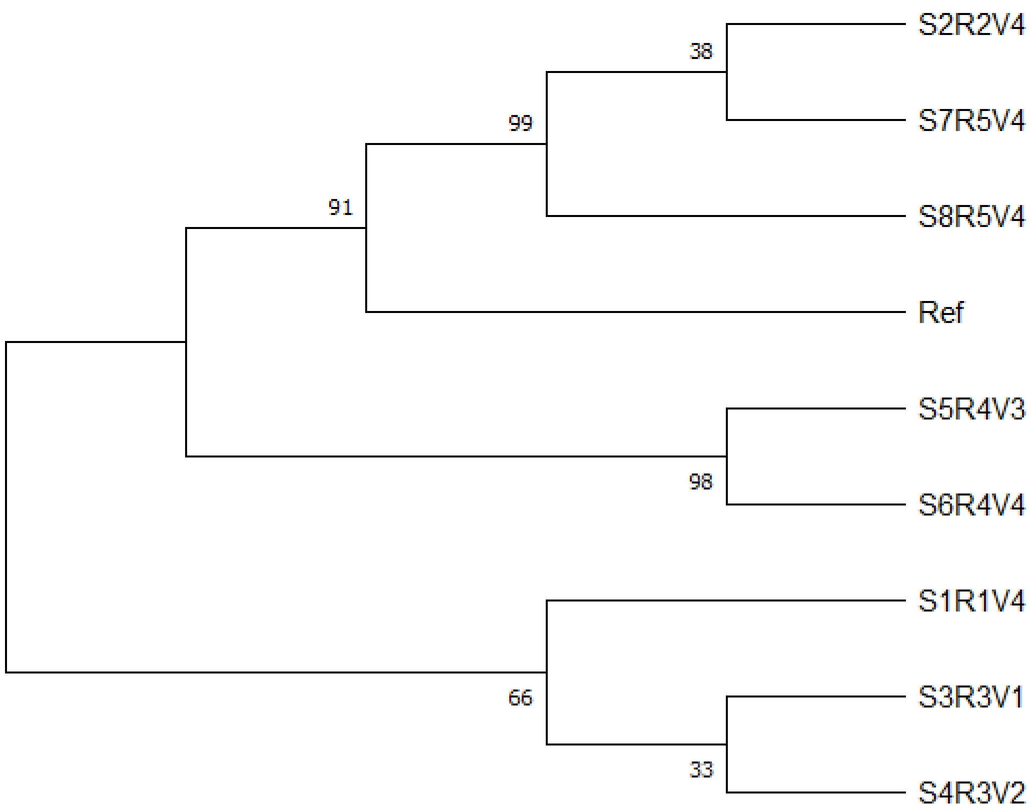

Figure S8. Maximum likelihood tree of *Lac 8* Gene sequences of *Botrytis cinerea* combined and compared for every strain. The strains selected were from different regions and vintages. Bootstrap values were calculated from 1000 iterations and are displayed in %.

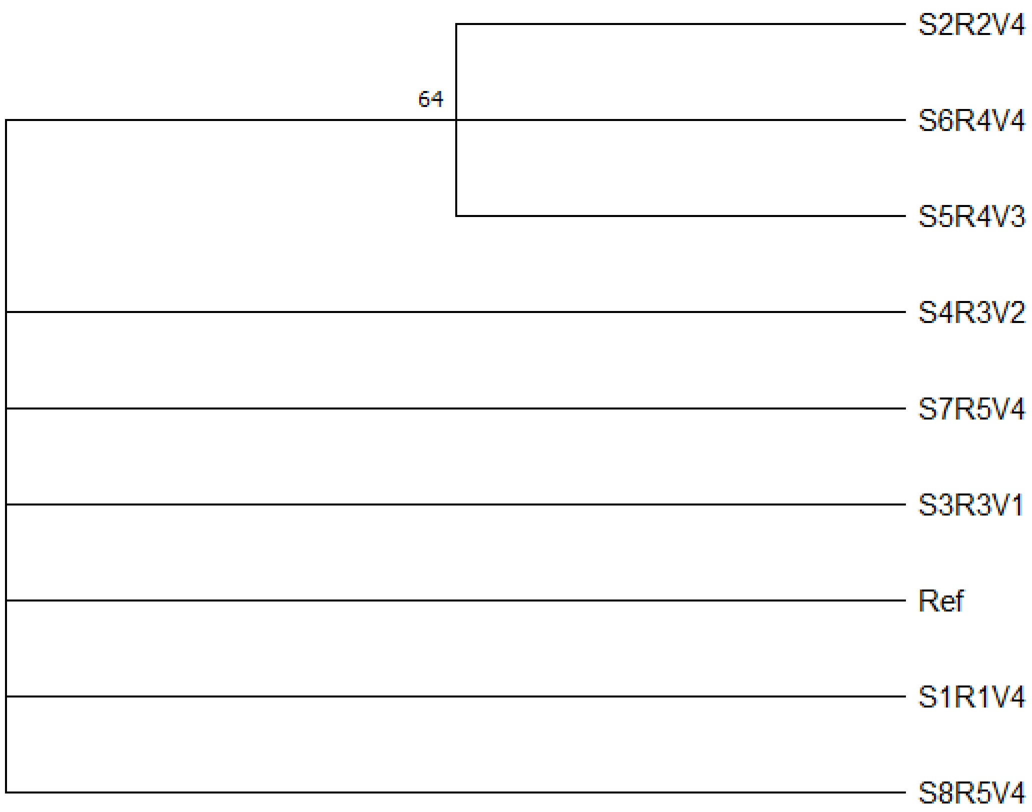

Figure S9. Maximum likelihood tree of *Lac 9* Gene sequences of *Botrytis cinerea* combined and compared for every strain. The strains selected were from different regions and vintages. Bootstrap values were calculated from 1000 iterations and are displayed in %.

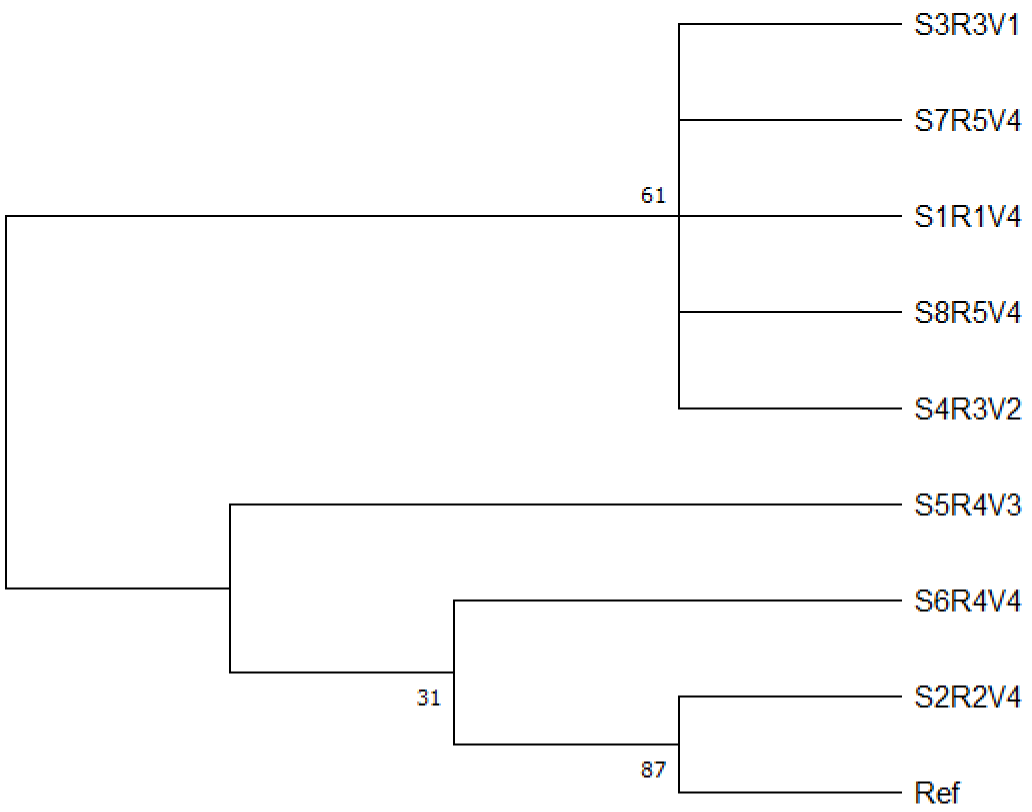

Figure S10. Maximum likelihood tree of *Lac 10* Gene sequences of *Botrytis cinerea* combined and compared for every strain. The strains selected were from different regions and vintages. Bootstrap values were calculated from 1000 iterations and are displayed in %.

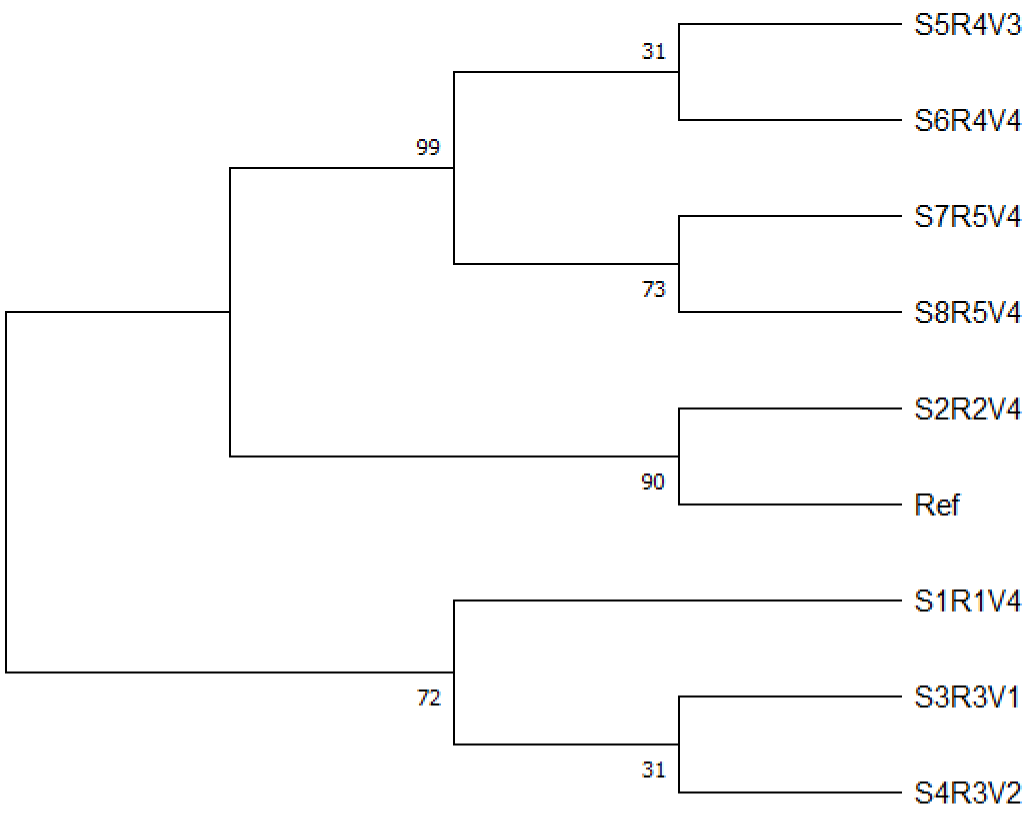

Figure S11. Maximum likelihood tree of *Lac 11* Gene sequences of *Botrytis cinerea* combined and compared for every strain. The strains selected were from different regions and vintages. Bootstrap values were calculated from 1000 iterations and are displayed in %.

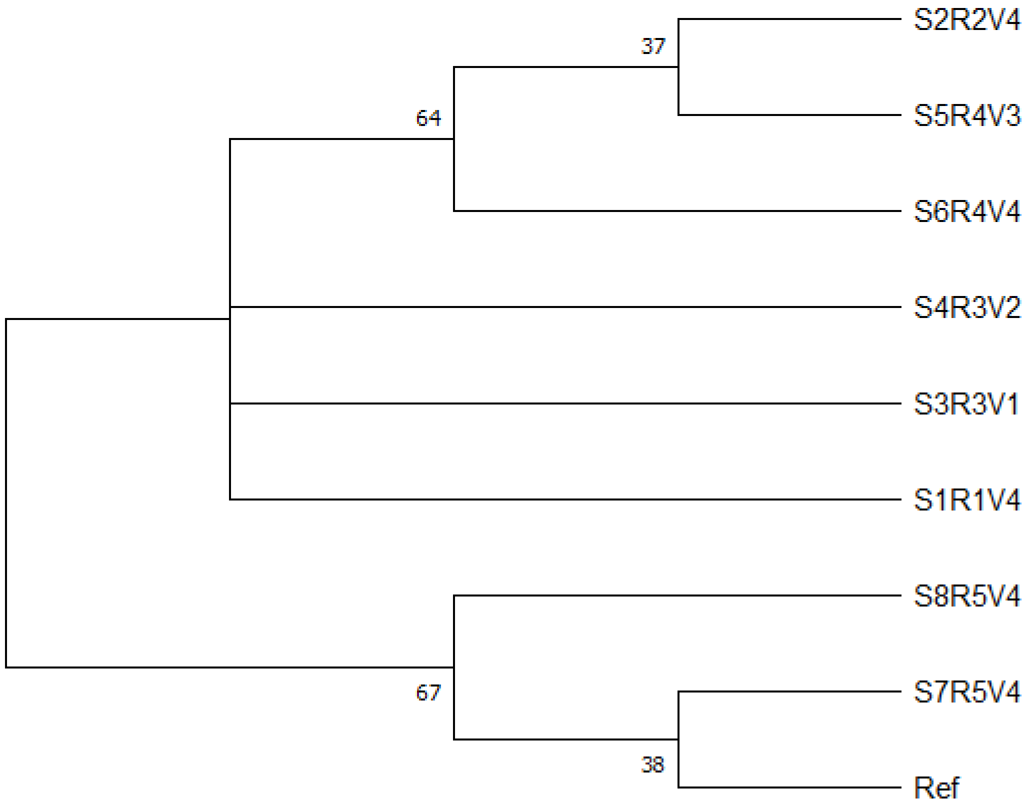

Figure S12. Maximum likelihood tree of *Lac 12A* Gene sequences of *Botrytis cinerea* combined and compared for every strain. The strains selected were from different regions and vintages. Bootstrap values were calculated from 1000 iterations and are displayed in %.

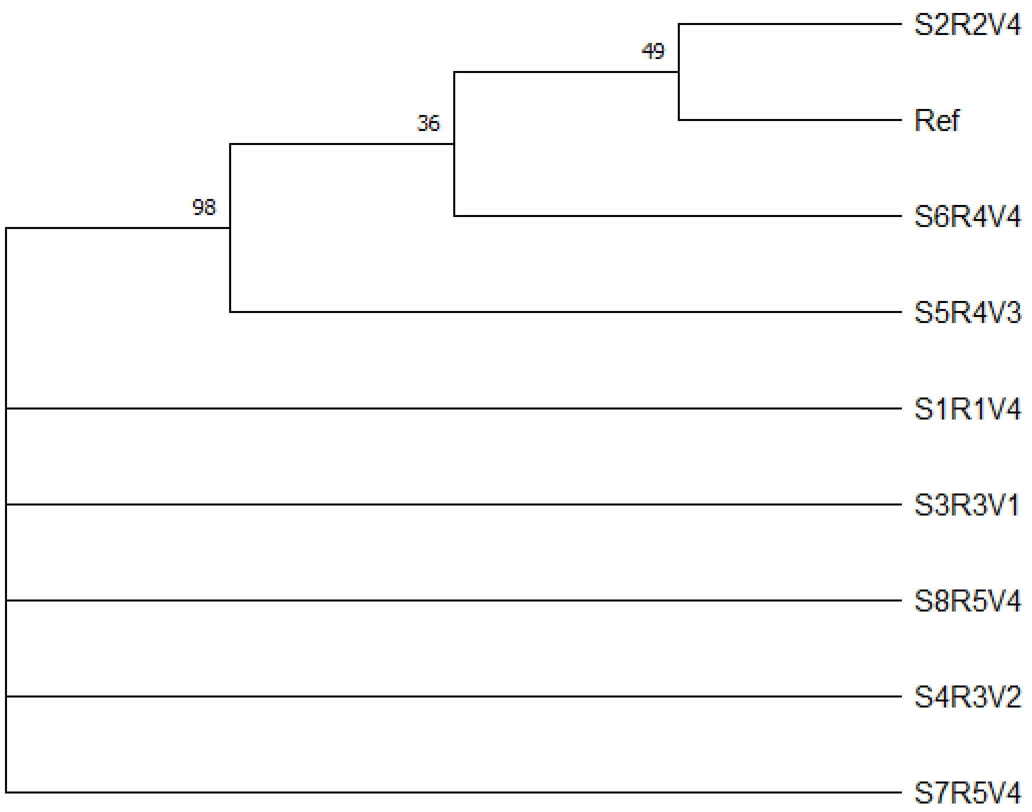

Figure S13. Maximum likelihood tree of *Lac 12B* Gene sequences of *Botrytis cinerea* combined and compared for every strain. The strains selected were from different regions and vintages. Bootstrap values were calculated from 1000 iterations and are displayed in %.

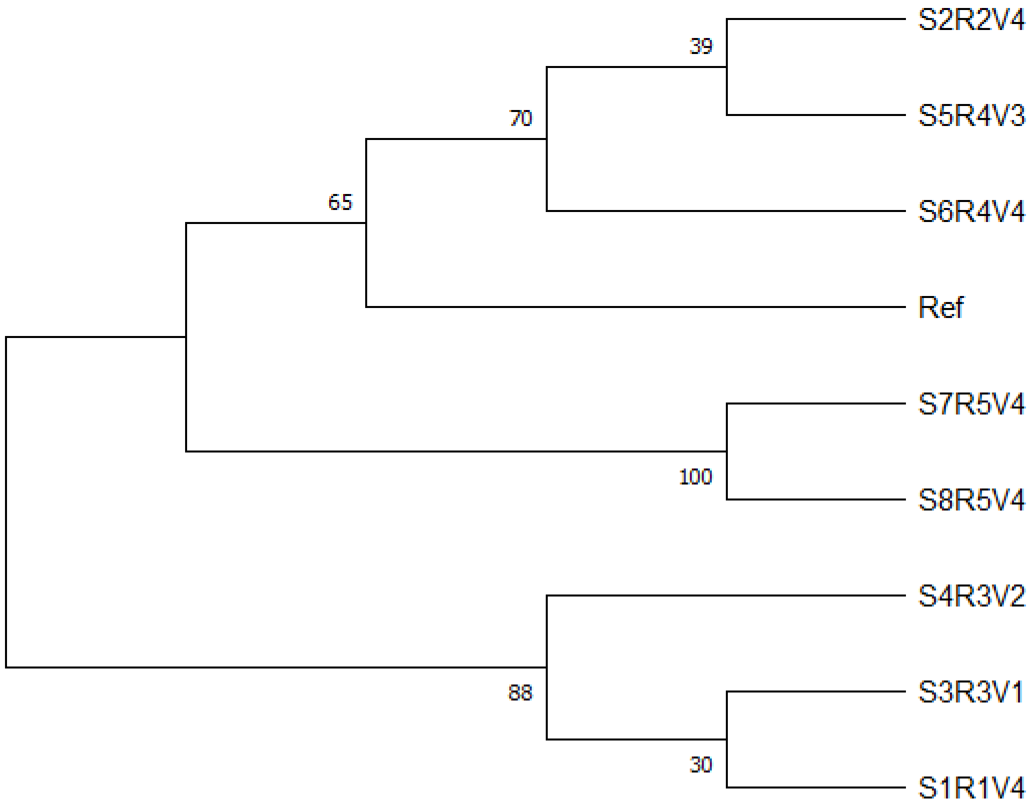

Figure S14. Maximum likelihood tree of *Lac 13* sequences of *Botrytis cinerea* combined and compared for every strain. The strains selected were from different regions and vintages. Bootstrap values were calculated from 1000 iterations and are displayed in %.

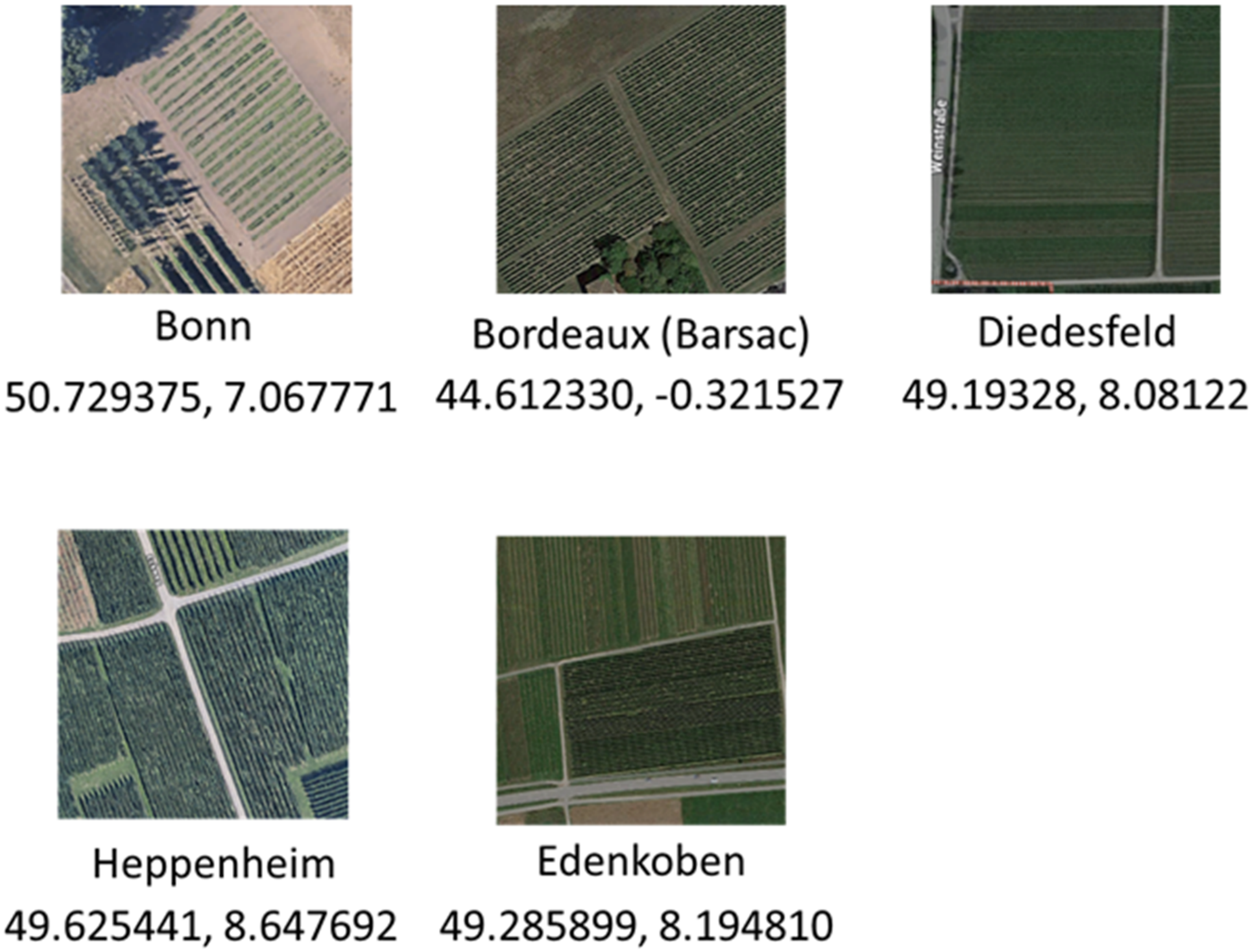

Figure S15. Satellite images of the collection sites. The images do not represent the vineyard at the collection time.
